# Antisepsis and Asepsis

**Published:** 1915-01

**Authors:** George H. Lee

**Affiliations:** Galveston, Texas


### Antisepsis and Asepsis.

It is interesting to review very briefly the development of the principles and their application in the production of surgical asepsis.

Sir David Lister after a lifetime of study, in which his results were much influenced by the work of Louis Pasteur on putrefaction and fermentation, arrived at the conclusion that infection resulted from the introduction of bacteria and was essentially a process of putrefaction. These organisms were introduced by contact of clothing, dressings, instruments, surgeons' hands, etc., but also were air borne. His efforts were directed to the sterilization of wounds by protecting those wounds from infection through the air and from contact by the application of antiseptic agents, chemicals, etc., i. e., carbolic acid. Wounds were painted with carbolic acid, then wrapped in lint which had been moistened with that agent. Operations were done in an atmosphere in which was playing a spray of carbolic acid. Instruments were prepared by immersing for fifteen minutes in 3 to 5 per cent carbolic acid. Chemical sterilization was attempted throughout, and remarkable progress followed.

At least the surgical world awakened. The bacteriological laboratory came into existence and demonstrated to the surgeon with some accuracy what method of sterilization was efficient, how instruments, sponges and dressings could be sterilized, when sterilization was inefficient and how most nearly efficient.

Toward the end of the period of constant use of iodoform, mercury bichloride, and other active chemical agents it was shown that the too full use of these agents frequently interfered with the satisfactory healing of wounds by inhibiting the factors in the living tissues that contributed to prompt physiological restoration to normal.

The next era, and to this day the most satisfactory, was that of sterilization by heat, with the laboratory culture inoculation check as to accuracy and efficiency. This method was developed to a point of demonstrable sterility in so far as were concerned dressings, instruments, sutures and ligatures, sponges, gauze, sheets, etc., used about the operating field and water. Unfortunately the patient could not be boiled. But more attention came to be devoted to the preservation of the living element of resistance in the tissues of the immediate field of operation. The skin and subdermal tissues could not be rendered sterile, but these could be cleansed surgically and the more superficial epithelial layers rendered sterile; the ability of those tissues to resist infection could be encouraged and preserved by gentle handling, by preservation of the blood supply, by not leaving dead space when the tissues were approximated, by limiting the number of punctures of the skin.

The hands of the operator and assistants proved the most difficult factors in this effort to produce an aseptic operation. These likewise could not be boiled. The nails and crevices of the skin resisted disinfecting and sterilizing methods. The yearning of the surgeon was for a boiled hand for himself and his assistants that could be revised by the bacteriologist and reported as not producing cultures. It came in the rubber glove, boiled and sterilized, combined with the previous soap and water and bichloride preparation of the hands, and careful technique in putting on the glove. How lamentable to see a would-be aseptic operator smooth out the fingers of the glove on one hand with the bare fingers of the other hand. As a new assistant once answered, "My hands have been sterilized," referring to the usual operating room methods. If sterile, why the rubber glove?

Heat sterilization has its objections, however. No surgeon is satisfied to use a dull knife or scissors. Heat interferes very materially with the cutting properties of such instruments. Therefore, the method of immersing knives and scissors in pure carbolic acid for ten minutes and then in 95 per cent alcohol, instead of boiling, is a return to chemical sterilization, but in a more efficient application.

This is not all. The doctrine has come into existence that the operative field can be efficiently prepared by what is known as the dry method; i. e., the application of benzine and iodine, or of iodine alone, a return to chemical sterilization in another form. The method has gained quite wide acceptance and is at the present time apparently giving satisfactory results. The future may render a different verdict. Several very carefully prepared articles based on experience, checked by laboratory experiment, have appeared in the medical journals; which have had for their object the giving of the results of the testing of this new method of preparation of the operative field, as contrasted with the former more elaborate preparation with green soap and sterile water, bichloride compresses, etc. In several of these articles the conclusion is reached that the application of iodine according to present methods does not sterilize, but by the presence of the iodine the development of the bacteria are simply inhibited, consequently we can feel certain that the end is not yet. The future must bring changes and increased perfection of technique.—Written for TEXAS MEDICAL JOURNAL by George H. Lee, M. D., Galveston, Texas.

